# Pregabalin Dependence and Management in a 55-Year-Old Female with Chronic Low Back Pain

**DOI:** 10.7759/cureus.44085

**Published:** 2023-08-25

**Authors:** Abhimanyu Vasudeva, Richa Tripathi

**Affiliations:** 1 Physical Medicine and Rehabilitation, All India Institute of Medical Sciences, Gorakhpur, Gorakhpur, IND; 2 Psychiatry, All India Institute of Medical Sciences, Gorakhpur, Gorakhpur, IND

**Keywords:** dependence, tapering, chronic pain, pain, pregabalin

## Abstract

This case report explores the complexities of managing chronic pain and the subsequent development of pregabalin dependence in a 55-year-old female patient with a prior history of vertebral fracture. Over a period of 10 years, the patient relied on a combination of Aceclofenac and pregabalin to alleviate her pain. An alternative treatment approach was implemented, involving adjustments to medication dosages and gradual tapering. Throughout the treatment process, interdisciplinary collaboration played a pivotal role in addressing unexpected symptoms such as facial movements and neck swelling. This case report highlights the significance of recognizing and addressing pregabalin dependence in patients with chronic pain.

## Introduction

Both gabapentin and pregabalin belong to the group known as gabapentinoids. Pregabalin, a gamma-aminobutyric acid (GABA) analog, belongs to the group of new-generation anticonvulsant drugs. Its mechanism of action involves the reduction of the release of glutamate, noradrenaline, and substance P. It is prescribed for many health conditions such as fibromyalgia, postherpetic neuralgia, and neuropathic pain [1]. Additionally, it finds applications as an off-label medication for various psychiatric conditions, including insomnia, obsessive-compulsive disorder, post-traumatic stress disorder, anxiety associated with schizophrenia, and in the treatment of benzodiazepine and alcohol use disorder [2,3]. Gabapentin, a gabapentinoid, holds the potential to assist in opioid abuse treatment as an adjunct therapy and might play a role in addressing cannabis dependence independently. However, the current evidence is inadequate, and its impact on cocaine and amphetamine abuse remains uncertain [4]. While administered at therapeutic doses, the probability of addiction is unlikely [1].

## Case presentation

A 55-year-old female patient with a history of an old fracture of the L2 vertebra presented to the Department of Physical Medicine and Rehabilitation of a tertiary healthcare facility with complaints of low back pain for the last 20 years, with radiation of pain to her right lower limb. For the past 10 years, the patient was on a combination of Aceclofenac (200 mg) and pregabalin (75 mg) at night to alleviate her pain. An additional dose of pregabalin (75 mg) was taken, also at night. Although initially prescribed for two weeks, she continued using them without further consultation, leading to her dependence on pregabalin.

She expressed concerns about the prolonged use of painkillers and desired to stop taking them. She had attempted in the past to reduce the dose by taking half a tablet or stopping it altogether; however, she was unsuccessful in this as on doing so, she experienced intense pain in her back, along with symptoms of irritability, sleeplessness, low mood, and decreased interest in her day-to-day activities. These symptoms suggested dependence. However, on the other hand, despite her pain not being completely relieved, over the years, she did not increase the dosage either and continued to take one tablet of combined Aceclofenac and pregabalin at night with the additional dose of pregabalin.

**Figure 1:**
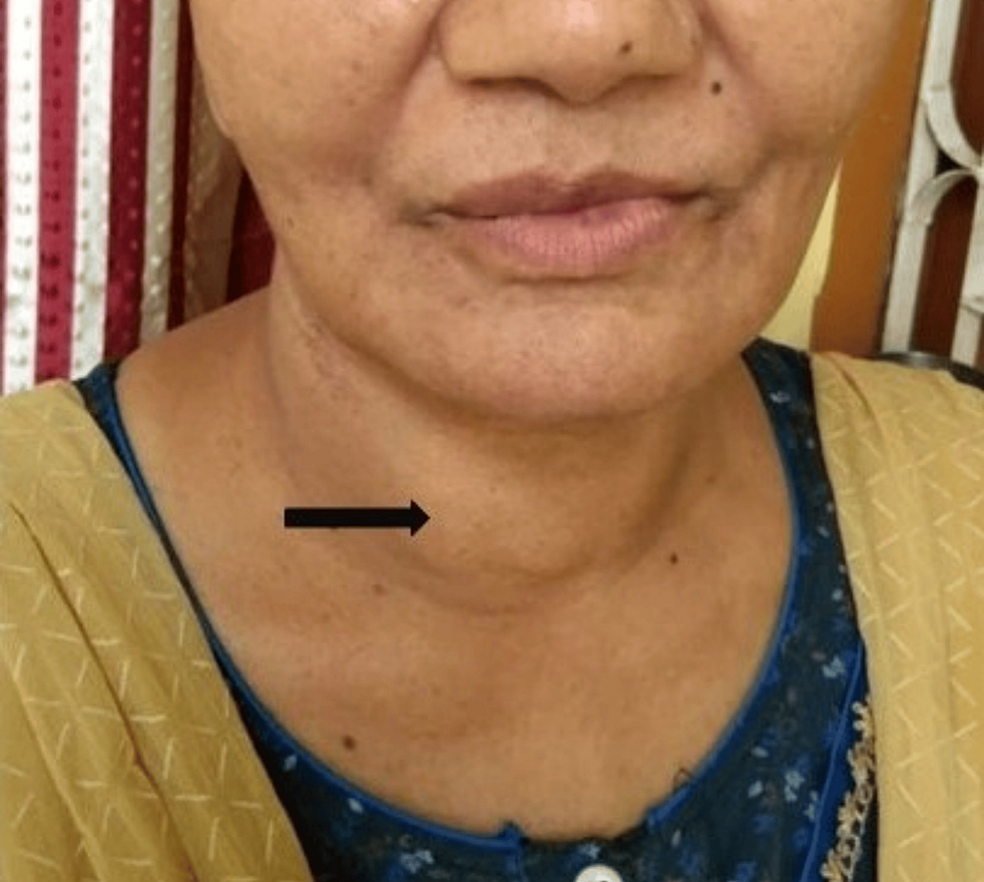
Incidental finding of swelling in the neck

After the initial assessment, she was advised the following investigations: complete blood count (CBC), kidney function test (KFT), liver function test (LFT), thyroid profile, blood sugar, serum calcium, and vitamin D, all of which were found to be within normal limits. During the general survey, we came across another clinical finding: a neck swelling (Figure 1) for which she was referred to the Department of ENT. Ultrasonography (USG) of her neck revealed colloid nodules. Subsequently, fine-needle aspiration cytology (FNAC) was suggested.

We introduced a new treatment regimen that included Aceclofenac (100 mg) and paracetamol (325 mg) on an as-needed basis, and pregabalin (75 mg) twice a day, which replaced the previous cumulative dose of 150 mg taken at night. The adjustment aimed to distribute the effects of pregabalin throughout the day. It was planned to taper the medicines gradually, and she was requested to follow up after a week.

After a week of follow-up, the patient and her informant reported symptoms of significant irritability and facial deviation/movements. A medicine consultation was taken to rule out focal seizures. She was also referred to the Department of Psychiatry for her irritability, sleep disturbance, facial movements, and unresolved pain complaints.

The patient had no previous history of mental illness. A diagnosis of pregabalin dependence was made, keeping in mind her constant desire to take the medicine, withdrawal symptoms in the form of dizziness, headache, irritability, restlessness, disturbed sleep, lack of interest in day-to-day activities, and continued use despite knowing the harmful effects of long-term use.

Aceclofenac was gradually tapered and discontinued over two weeks, while pregabalin was tapered down to a nightly dosage of 75 mg, which was maintained. During the tapering process, the patient was initiated on clonazepam 0.5 mg for her sleep complaints and restlessness, which was continued for four weeks. The patient demonstrated a positive response to the gradual tapering approach with concurrent use of clonazepam. Currently, the patient continues on pregabalin at a nightly dosage of 75 mg.

The patient was also evaluated for other depressive and anxiety symptoms, but she denied having such symptoms. The family member observed that her irritability gradually subsided after starting clonazepam. Although she appeared unconcerned about her neck swelling, that might be another cause of her irritability.

## Discussion

This case study illustrates the difficulties inherent in managing chronic pain over an extended period. It draws attention to the profound significance of tailoring treatments to individual needs and fostering collaborative efforts among various disciplines. The unexpected emergence of new symptoms coupled with subsequent adjustments in medication highlights the intricate nature of pain management. This emphasizes the critical nature of vigilant observation and the need for flexibility to modify treatment strategies.

According to a systematic review by Bonnet and Scherbaum, the risk of developing dependence on gabapentinoids remains very low among patients without a history of substance use disorders [5]. Another review by Hofmann and Besson emphasizes that it is crucial to consider the likelihood of their improper use when considering the administration of such medications [6].

The utilization and potential improper consumption of pregabalin are reported to be raising concerns, particularly in the western region of Punjab, India [7]. Furthermore, another study suggests that while the inherent addictive tendencies of these medications aren't supported by current evidence, caution must still be exercised due to the potential for abuse among individuals with opioid use disorders [8]. This highlights the necessity of a comprehensive risk assessment.

A systematic review by Evoy et al. suggests that there is a growing trend of gabapentinoid misuse and abuse, resulting in harm to patients [9]. Healthcare providers should thus exercise caution, particularly when dealing with high-risk patients, and remain vigilant for any signs of misuse or abuse. However, Bonnet et al, in response to the aforementioned review, bring attention to the lack of research focusing on individuals who do not have previous or current substance use issues [10]. Furthermore, they emphasize the importance of having solid evidence from well-conducted studies before claiming potential harms and addictive qualities of these substances . This reiterates the importance of a nuanced and comprehensive understanding of the complexities surrounding gabapentinoid usage.

Starting from April 2019, the classification of gabapentin and pregabalin has been changed in the United Kingdom to 'controlled substances' [11]. Pregabalin has been categorized as a Schedule V drug in the United States [12]. Schedule V drugs are the least likely of the controlled substances to be misused. A study by Peckham et al. puts forth a call for the federal-level reclassification of gabapentin, which currently maintains its status as an uncontrolled substance [13]. However, several states within the United States have implemented specific measures to address the concerns of diversion and abuse.

## Conclusions

This report highlights the challenges of managing chronic pain and the significance of tailored treatments. Pregabalin's usage and potential for patient dependence were examined, along with effective tapering strategies. The importance of interdisciplinary collaboration in pain management was emphasized. Gabapentinoids seem to have a low addiction risk in non-substance users, although concerns about pregabalin misuse persist. The report advocates personalized care and collaboration across disciplines and casts light upon the intricate nature of treatment adjustments and the necessity of vigilant observation. The findings contribute to the ongoing discourse surrounding gabapentinoid usage and potential risks.
